# Designing a Universal Glass Composite for Plaster Mortars

**DOI:** 10.3390/ma19112312

**Published:** 2026-05-29

**Authors:** Wiktor Szewczenko, Galyna Kotsay

**Affiliations:** Faculty of Civil Engineering, Mechanics and Petrochemistry, Warsaw University of Technology, Łukasiewicza St. 17, 09-400 Płock, Poland; wiktor.szewczenko@pw.edu.pl

**Keywords:** water glass, glass composite, adhesive strength, waste glass

## Abstract

Currently, construction uses a vast array of materials that, while serving the same purpose, differ only slightly in their properties. This complicates the substitution of one material for another, significantly expanding the product range when considering operating conditions, necessitating expanded warehouse space. Therefore, preference should be given to universal materials that, while maintaining the same chemical composition, can change their properties by altering the ratio of their components. This study addresses this issue by evaluating the potential of glass composites containing powdered waste glass as alternatives to selected conventional construction materials. The results demonstrated that the rheological properties of the composites can be effectively controlled by adjusting the ratio of water glass to waste glass powder, enabling the achievement of viscosity values suitable for both plastering and installation mortars. In addition, the composites exhibited markedly higher adhesion strength than conventional gypsum mortars under high-humidity conditions, confirming their applicability as adaptable, substrate-specific materials with geopolymer-like characteristics.

## 1. Introduction

In construction practice, plastering mortars constitute a group of materials with a diverse composition, differing in the type of binder, aggregate, and the additives and admixtures used [1,2,3,4,5,6]. For interior finishes, gypsum plasters or plasters based on gypsum binders compliant with PN-EN 13279-1 standard [7] are commonly applied, as well as cement–lime plasters compliant with PN-EN 998-1 [8]. According to EN 15824 standard [9], interior plasters may also include those based on organic binders, as well as silicate- and clay-based plasters. The selection of an appropriate plaster is determined by the functional requirements of the room, moisture conditions, and the type and condition of the substrate. Increasingly, “universal” interior plasters with formulations modified by additives are also used to stabilize application parameters and to improve tolerance to varied substrates and execution conditions.

Gypsum plasters are predominant in dry indoor environments due to the high quality of the finished surface, good workability, and high application efficiency, particularly when applied by machine spraying [10,11]. Cement–lime plasters are more commonly used in areas exposed to elevated moisture and higher loads, where increased resistance to water action and mechanical damage is required [2,12,13]. Proper substrate preparation is essential to achieve the required plaster adhesion; this is particularly important for gypsum plasters, which are highly sensitive to substrate condition and variations in surface absorbency. In addition, for gypsum plasters, excessively rapid carbonation may lead to tensile stress development within the plaster–substrate system due to an increase in solid-phase volume (≈11.6%) and to water formation during carbonation, which can soften gypsum plaster and thereby reduce adhesion at the plaster–substrate interface. Cement–lime plasters, in turn, exhibit higher resistance to moisture and greater mechanical strength; however, they typically show higher shrinkage, which may induce stresses within the plaster layer and increase the risk of cracking or local debonding [14,15,16,17,18].

Another less commonly used group comprises silicate plasters, which in practice are most often applied on building exteriors, particularly as thin coat finishes in ETICS (External Thermal Insulation Composite Systems). In interior applications, silicate-based solutions are more frequently used in the form of silicate paints or renovation systems rather than as conventional plasters [19]. Silicate plasters belong to the group of mineral plaster finishes characterized by good compatibility with mineral substrates, high water vapor permeability, and favorable resistance to atmospheric exposure. The primary binder used in this type of material is potassium water glass, which contributes to hardening through a silicification reaction with the mineral substrate and carbonation of silicate components. Quartz sands and quartz flour are most commonly used as mineral constituents, along with fine carbonate fillers such as calcite or dolomite. Their particle size distribution is selected depending on the required layer thickness, surface texture, and rheological properties of the mortar. To ensure appropriate application properties, mixture stability, and control of the hardening process, modifying additives and admixtures, including rheology-control agents, stabilizers, defoamers, and setting regulators, are incorporated to optimize workability, prevent segregation and excessive water loss [20,21,22,23].

Silicate binder exhibits a different hardening mechanism compared with gypsum and cementitious binders. Whereas the setting of gypsum and cementitious binders is primarily associated with hydration reactions, the setting and hardening of water glass occur mainly through silicate polycondensation [24,25]. The different setting mechanism and the type of products formed during this process may result in silicate binders exhibiting higher adhesion to mineral substrates than gypsum and cementitious plasters [26]. However, a significant challenge associated with the use of silicate binders is the viscosity of the paste. Since the workability of plasters largely depends on the consistency of the mortar, the appropriate selection of component proportions is crucial for obtaining a material with the desired application properties. The aim of this study is to use waste glass in silicate plasters and to develop a silicate plaster formulation with a viscosity comparable to that of gypsum and cementitious plasters.

## 2. Materials and Methods

Sodium and potassium water glass produced by Chemical Plant “Rudniki” S.A. (Rudniki, Poland) [27], as well as industrial waste glass provided by REWA (Koluszki, Poland) [28], were used as source materials for the glass composite (GC). Their composition and properties are presented in Table 1. The modulus and density of the water glasses were determined using the methods described in standards [29,30]. The density and BET specific surface area of the waste glass were determined according to the methods described in the relevant standards [31,32].

The rheological measurements (dynamic viscosity) were conducted using the IKA ROTAVICS me-vi (Staufen in Breisgau, Germany). An SP-8 spindle was used for all the samples. The paste water glass and waste glass were prepared in a mixer. Mixing time—90 s, 30 s at low speed and 60 s at high speed. The measurement of rheological parameters was conducted 5 min after the completion of mixing the components. The temperature during the test was maintained at a constant level of 20 °C.

The study of thermal effects of chemical reactions between the components of the glass composite was carried out using a Calmetrix I-Cal 2000 HPC isothermal calorimeter (Boston, MA, USA) [29].

The following materials were used as substrates: sand–lime brick manufactured by PREFABET Osława Dąbrowa S.A. (Osława-Dąbrowa, Poland) [33] and dry-pressed ceramic tiles manufactured by Cersanit (Kielce, Poland) [34]. Adhesion of glass composites to the substrate was determined using a specially developed method. The test material was applied to the substrate surface as a 4 mm thick layer. The adhesion strength was determined based on four individual specimens, and the reported values are presented as the mean ± standard deviation of four measurements. After 7 days of storage at room temperature, a 50 mm diameter circle with a depth of 5–6 mm was cut into the surface. Metal disks with a threaded center were then glued to the surface of the circles for connection to a Pull of DY-2 apparatus (Proceq SA, Schwerzenbach, Switzerland). The adhesion results are presented in MPa. Adhesion strength was determined on four individual specimens for each composite, and the results are reported as mean values ± standard deviation. All the studies conducted on the above-mentioned equipment were carried out in accordance with European standards [35].

## 3. Results

The European market offers a wide range of building mortars based on natural and synthetic gypsum, cement, and cement–lime binder systems, typically designed for plastering, masonry, and installation applications. However, universal mortars have recently emerged that can be used for both types of work, as shown in Table 2.

The most important parameter for all mortars is the water demand, which corresponds to the working viscosity. As shown in Table 2, despite differences in water demand, the viscosity of the gypsum mortars remains around 63 Pa·s; only for the universal mortars does it range from 25 Pa·s to 71 Pa·s. Therefore, taking the viscosity corresponding to the working consistency of the gypsum mortars presented in Table 2 as a reference, it is possible to obtain mortars with comparable consistency in a different system, namely a powdered glass waste–water glass system, hereinafter referred to as a glass composite. By determining the relationship between the viscosity of the glass composite and the ratio of its components, it is possible to select a composition that corresponds to the working consistency of the mortars presented in Table 2.

The method for designing glass composite (GC) compositions discussed in this paper is based on determining the ratio of waste glass powder to water glass using the dependence of glass composite viscosity on the ratio of water glass to glass powder.

**Figure 1:**
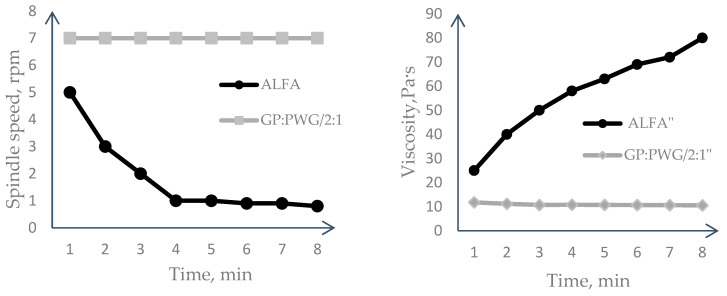
Dependence of spindle speed and viscosity on time for ALFA gypsum mortar and GP:PWG/2:1 glass composite.

Given the significant importance of viscosity and its relationship to working consistency, specific viscosity studies of various mortars were conducted, taking into account that all the systems under consideration were classified as non-Newtonian fluids [41]. Since virtually all mortars change their viscosity over time, a process known as setting time in construction technology, the time interval from the moment the components are mixed to the moment the viscosity stabilizes on the viscometer should be determined. Experiments have shown that in the viscosity range studied, this time interval is 6.5 min. A total of 5 min of this time is allocated to stabilize the operating mode of the viscometer. This is evidenced by the stabilization of the spindle speed after 5 min of operation, as shown in Figure 1.

Considering the fact that the slowdown in spindle speed indicates thickening of the ALFA gypsum mortar during the studied time period, while for the glass composite it is stable, it can be assumed that the hardening rate of the glass composite at the initial stage is much lower compared to the gypsum mortar. This is confirmed by the results of determining the viscosity of the aforementioned substances, presented in Figure 1. The correlation coefficient between viscosity and spindle speed (Figure 1) is −0.93, indicating a decrease in spindle speed as the viscosity of the mortar increases. The spindle load (>50%) confirms the accuracy of the measurements. It should be emphasized that the glass composite exhibits virtually no change in viscosity during the studied time (8 min), unlike the gypsum mortar.

**Figure 2:**
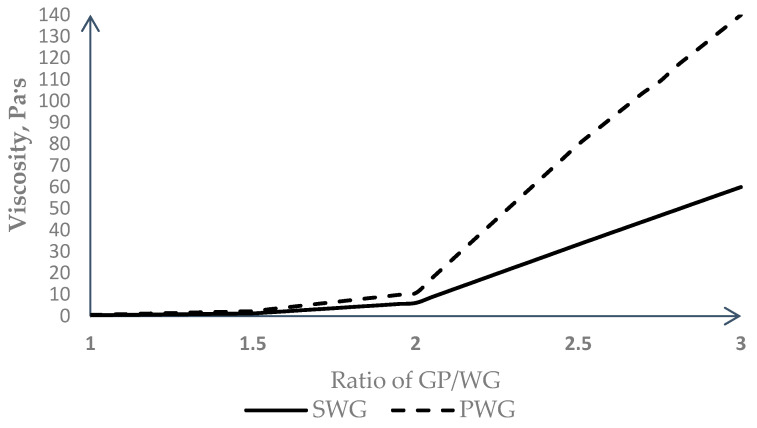
Dependence of the viscosity of glass composites on the ratio of glass powder and water glass.

As studies have shown, the viscosity of the glass composite depends to a large extent on the ratio of the adhesive substance, in our case water glass, and the filler—waste glass in the form of powder [42]. Figure 2 shows the dependence of the viscosity of the glass composite on the GP/WG ratio.

The graphical relationship presented in Figure 2 can be divided into three zones: (1) GP/WG from 1.0 to 1.5, (2) GP/WG from 1.5 to 2.0, and (3) GP/WG from 2.0 to 3.0. The first and second zones are characterized by low viscosity values, which fall outside the range of viscosities presented in Table 1; therefore, they will not be considered further. The third zone is of greatest interest, as it includes the viscosity values corresponding to the practical mixtures presented in Table 1. Thus, by adopting the viscosity value of a given mixture from Table 1, corresponding to the working consistency, it appears possible to determine the GP/WG ratio from the graphical relationship shown in Figure 2 and, on this basis, to calculate the composition of the designed glass composite. The proposed design method is illustrated below using specific examples.

As shown in Table 2, the viscosity of all the gypsum-based mortars ranges from 63 Pa·s. By selecting the value of 63 Pa·s, it can be seen from Figure 1 that this viscosity corresponds to a GP/PWG ratio of 2.38. Based on this, the percentage composition of the designed glass composite is as follows: GP—70.4% and PWG—29.6%. Accordingly, for a universal mortar with a working consistency corresponding to a viscosity of 71 Pa·s, the glass composite composition is: GP—70.8% and PWG—29.2%. Thus, a difference of 8 Pa·s results in a change in the amount of binder and filler (glass waste) of 0.4%. It should be emphasized that such a slight difference in viscosity is typical of mixtures used solely as plaster mortars. In the case of a universal mortar used for both installation and plastering works, a viscosity of 25 Pa·s is required to achieve the appropriate working consistency (composition No. 4, Table 2), which corresponds to the following composition: GP—67.6% and PWG—32.4%. In this case, the differences in composition amount to 2.8% and 3.2% relative to the glass composites with viscosities of 63 Pa·s and 71 Pa·s, respectively.

**Figure 3:**
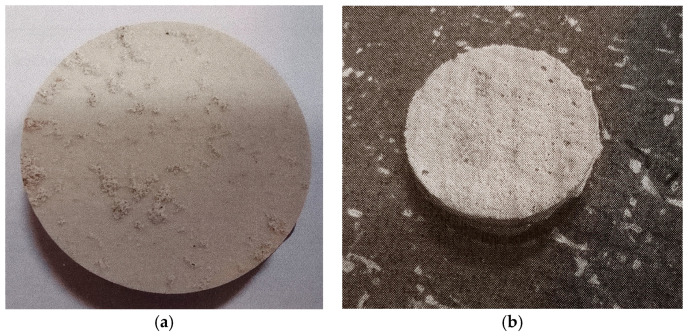

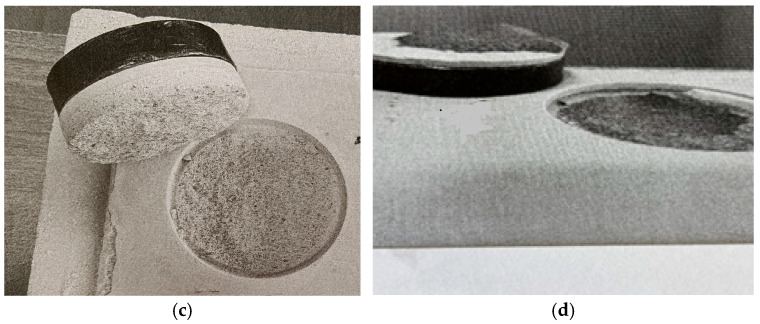
Surface of the glass composite after separation from the substrate surface: (**a**)—f_adh_ to substrate sand–lime brick; (**b**)—f_adh_ to substrate ceramic tile; (**c**)—f_coh_ to substrate sand–lime brick; and (**d**)—f_coh_ to substrate ceramic tile.

Due to the difference in viscosity between sodium and potassium water glasses (Figure 3), there is a significant difference in the designed compositions of potassium and sodium glass composites. For example, for universal mortar N4 (Table 1), the composition of the sodium glass composite is GP—70.2% and SWG—29.8, while that of the potassium PWG is GP—67.6 and PWG—32.4. It should be emphasized that a working consistency of 63 Pa·s cannot be achieved for the sodium glass composite (Figure 1).

Table 3 shows the designed glass composite compositions, which, in terms of working consistency (viscosity), correspond to the gypsum mortars presented in Table 1.

The composition of the glass composite significantly influences its rheological and mechanical properties. An increase in the proportion of water glass relative to glass powder leads to a noticeable decrease in dynamic viscosity. Lower viscosity improves the workability of the composite and enhances its ability to wet and penetrate the substrate surface. As a result, composites with lower viscosity exhibit higher adhesion strength. The experimental results indicate that reducing viscosity from 71 Pa·s to 25 Pa·s increases adhesion from approximately 0.8 MPa to over 1.1 MPa. Furthermore, the type of water glass used plays a crucial role in determining the final properties of the composite. Sodium water glass provides significantly higher adhesion compared to potassium water glass, reaching values above 1.6 MPa. This behavior is likely related to differences in chemical composition, reactivity, and the structure of the formed gel. Composites based on sodium water glass also show higher density, which suggests the formation of a more compact microstructure.

Considering that the construction process and operation of building structures can occur under varying temperature and humidity conditions, the adhesion of mortars to various substrates (the bases onto which the mortars are applied) is of great importance. As mentioned above, in this case, the substrates used were sand–lime brick with a porosity of 15–25% and ceramic tiles with a porosity of 0.5–3.0%. This difference, in accordance with the mechanical theory of adhesion, suggests that in the case of sand–lime brick, mechanical adhesion will prevail compared to ceramic tiles (Figure 3).

Relatively high values of adhesion strength for ceramic tiles with significantly lower porosity compared to sand–lime brick may indicate the predominance of the process of chemical interaction at the phase boundary [43]. In this case, mechanical adhesion will largely depend on the viscosity of the mortar. In this case, preference should be given to glass composites of the first and second zones (from the ratio WG:GP/1:1 to 1:1.5 (Figure 3), which are characterized by a viscosity of 1–2 Pa·s. Such solutions are preferable to use as adhesives, for example, for the installation of foam plastic boards, when installing walls made of sand–lime brick.

To assess the role of chemical interaction between the substrate and the mortar (glass composite), specialized microcalorimetric studies were conducted. For this purpose, the substrate material (lime–lime brick and ceramic tile) was ground to a grain size of 0.125–0.25 mm, which, after mixing with the appropriate soluble glass, was placed in a microcalorimeter.

**Figure 4:**
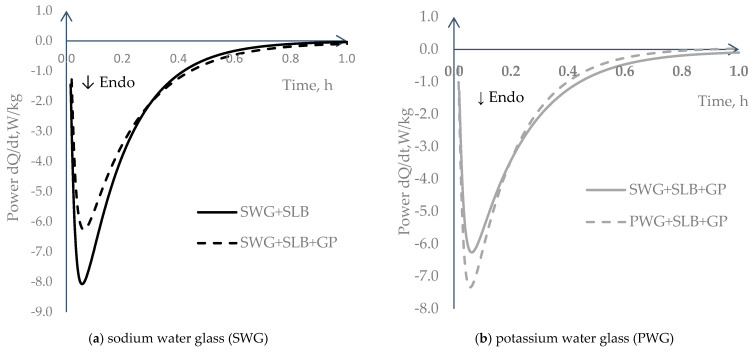

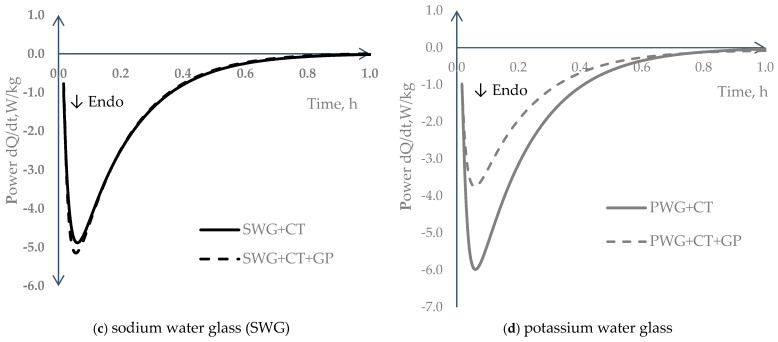
Dependence of the rate of heat release on time at the initial stage of dissolution when using sand–lime brick (SLB) (**a**,**b**) and ceramic tiles (CT) (**c**,**d**) as a substrate.

As is known [42,44], water glass acts as a binder in glass composites. It is its interaction with the substrate material that determines the adhesion strength of the glass composite. Therefore, the ability of water glass to react with the substrate material and the influence of glass waste on this process were studied (Figure 4).

The maximum endo effect for all the samples occurs approximately 2 min after adding water glass to the fillers. A sharp decrease in dQ/dt is observed for sodium (−8 W/kg) and potassium (−6 W/kg) water glasses. The nature of the curve in Figure 4 indicates that intensive dissolution of the fillers is taking place. In the case of sand–lime brick, this may be tobermorite (Ca_5_Si_6_O_16_(OH)_2_·4H_2_O), and in the case of ceramic tiles, mullite (3Al_2_O_3_/2SiO_2_). In a highly alkaline environment of water glass (pH ~12–13), tobermorite and mullite dissolve with the release of SiO_2_ in the ionic solution. Sodium water glass exhibits higher reactivity compared to potassium water glass. Additions of glass powder contribute to an increase in the amount of SiO_2_ in the ionic solution, as it is known [45,46,47,48,49] that inorganic glasses are capable of decomposing in an alkaline environment. In addition, glass powder serves as an additional source of alkaline cations, increasing the pH of the ionic solution. After reaching a minimum, the curves in Figure 4 tend to zero, indicating a slow attenuation of the chemical reactions and their transition to the stage of gel restructuring C-S-H → N-A-S-H or K-A-S-H, which is formed by thickening of the ionic solution. Moreover, systems with potassium-soluble glass reach equilibrium faster than those with sodium-soluble glass.

## 4. Conclusions

This paper presents a method developed by the authors for calculating the composition of a universal glass composite, which can be used as a mortar for installation and plastering work in construction. The selection of mortar consistency is based on determining the viscosity, which is 60–70 Pa s for plastering and 25 Pa s for installation work. By adjusting the ratio of water glass to waste glass powder, the desired mortar consistency can be achieved. The paper notes that the use of sodium water glass is limited due to its lower viscosity compared to potassium soluble glass. Replacing currently used gypsum mortars with glass composite mortars allows for a several-fold increase in adhesion strength under high-humidity conditions during application and operation. Studies of the adhesion mechanism have shown that the adhesion strength of glass composite to the substrate is determined by mechanical and chemical bonding mechanisms. The mechanical bonding mechanism is more prevalent in the case of a more porous substrate (sand–lime brick), while the chemical bonding mechanism is more prevalent in the case of a low-porosity substrate (ceramic tile). In the latter case, sodium water glass, which has a lower viscosity than potassium water glass, is preferable. Thus, by selecting the appropriate glass composition, it can be adapted to the specific operating conditions with maximum effectiveness.

Considering that chemical interactions occur when water glass and glass powder come into contact, involving dissolution processes and the formation of new reaction products, it may be assumed that the glass composite exhibits geopolymer-like characteristics. The developed glass composites can be considered promising materials for plastering and installation applications, particularly where enhanced adhesion is required. Future research will focus on the environmental durability of these composites. The developed glass composites can be considered promising materials for plastering and installation applications, particularly where enhanced adhesion is required. Future research will focus on the environmental durability of these composites.

## Figures and Tables

**Table 1:** Characteristics of sodium and potassium water glass and fine-ground glass waste.

Glass Materials	Oxides [wt%]				Density [g/cm^3^]	Modulus	Specific Surface [m^2^/g]
	Na_2_O	K_2_O	SiO_2_	H_2_O			
Sodium water glass (SWG)	8.02	-	26.38	65.6	1.34	3.4	-
Potassium water glass (PWG)	-	7.59	21.41	71.0	1.25	4.4	-
Waste glass powder (GP) *	10.50	4.50	72.00	-	2.43	-	0.0898

* The chemical composition of the waste glass was reported according to the manufacturer’s declaration (other oxides in the glass: R_2_O_3_, 1.0%; CaO + MgO, 12.0%).

**Table 2:** Examples of building mortars used in construction work.

Properties	Machine–Applied Gypsum Plaster ALFA [36]	Gypsum Filler CEKOL GS-100 * [37]	Gypsum Finishing Filler CEKOL C-45 GOLD [38]	Masonry and Plastering Mortar CEKOL ZMT-20 [39]	Cement Plastering Mortar HUZAR [40]
Composition of the plaster	CaSO_4_ >50% CaCO_3_ 30–40 Ca(OH)_2_ <5%	CaSO_4_ 60–75 CaCO_3_ 25–35 Ca(OH)_2_ <0.9%	CaSO_4_ 35–40 Dolomite 50–65	Cement 70–90 Sand 70–90 CaCO_3_ 10–13 Ca(OH)_2_ 1–3%	Cement <20% Sand <70% Ca(OH)_2_ <10 Fine <2%
Viscosity * [Pa·s]	63.0	63.1	63.2	25.0	71.0
Water requirement [L/kg]	0.64	0.45	0.40	0.14	0.20
Adhesion to the base [МPа]	>0.10	>0.50	>0.50	>0.30	>0.25
Сompressive strength [МPа]	>3.0	>3.0	>2.0	-	-
Density [g/cm^3^]	0.90	1.60	1.75	2.10	1.50

* The properties according to the authors.

**Table 3:** Compositions and properties of designed glass composites.

N	Compositions	Compound [%]			Dynamic Viscosity [Pa·s]	Density of Fresh Composite [g/cm^3^]	Fluidity of Fresh Composite [1/Pa]	Kinematic Viscosity [St]	Adhesion Strength to [MPa]	
		GP	PWG	SWG					Sand–Lime Brick	Ceramic Tiles
1	GP/PWG = 2.42	70.8	29.2	-	71.0	1.96	0.01	36.22	0.80 ± 0.05	0.80 ± 0.03
2	GP/PWG = 2.38	70.4	29.6	-	63.0	1.81	0.02	34.81	0.82 ± 0.06	0.80 ± 0.03
3	GP/PWG = 2.08	67.6	32.4	-	25.0	1.85	0.04	13.51	1.11 ± 0.07	1.08 ± 0.04
4	GP/ SWG = 2.35	70.2	-	29.8	25.0	2.12	0.04	11.79	1.63 ± 0.06	1.62 ± 0.05

## Data Availability

The original contributions presented in the study are included in the article. Further inquiries can be directed to the corresponding author.

## Abbreviations

The following abbreviations are used in this manuscript:

SWG	Sodium water glass
PWG	Potassium water glass
GP	Waste glass powder
GC	Glass composite
